# Opioid analgesic and antidepressant use during pregnancy and the risk of spontaneous preterm birth: A nested case–control study

**DOI:** 10.1111/ppe.13142

**Published:** 2024-11-17

**Authors:** Maria C. Padilla‐Azain, Sarah S. Osmundson, Olivia Bosworth, Andrew Wiese, Amelie Pham, Ashley A. Leech, Andrew J. Spieker, Carlos G. Grijalva, Margaret A. Adgent

**Affiliations:** ^1^ Department of Health Policy Vanderbilt University Medical Center Nashville Tennessee USA; ^2^ Department of Obstetrics and Gynecology Vanderbilt University Medical Center Nashville Tennessee USA; ^3^ Vanderbilt University Nashville Tennessee USA; ^4^ Department of Biostatistics Vanderbilt University Medical Center Nashville Tennessee USA

**Keywords:** antidepressants, opioids, pharmacoepidemiology, pregnancy, preterm birth, SSRIs

## Abstract

**Background:**

Given the high prevalence of both mental health and acute pain conditions during pregnancy, use of antidepressants and analgesic opioids in this period is widespread. Whether single and combined use of these medications is associated with spontaneous preterm birth (sPTB) remains unclear.

**Objectives:**

To investigate the association between maternal prescription opioid and antidepressant medication exposures for co‐occurring mental health and acute pain management, either alone or in combination, and sPTB.

**Methods:**

We used Tennessee Medicaid data (2007–2019) linked to birth certificates to conduct a nested case–control study among 15‐ to 44‐year‐old pregnant patients with singleton live births. Cases were identified as spontaneous live births between 24 and <37 gestational weeks using a validated birth certificate‐based algorithm. We selected up to 10 controls per case, matched on estimated pregnancy start date and other factors. We identified analgesic opioid and antidepressant pharmacy fills to define medication exposures in the 60 days before index date (case delivery date) and categorised them as unexposed, opioid‐only, antidepressant‐only and combined exposure. We estimated odds ratios (OR) and 95% confidence intervals (CI) using conditional logistic regression, adjusting for confounders. We assessed the additive interaction between opioids and antidepressants by estimating relative excess risk due to interaction.

**Results:**

We identified 25,406 eligible cases of sPTB and 225,771 matched controls. Opioid‐only and combined exposures were associated with higher odds of sPTB relative to unexposed (adjusted OR 1.29, 95% CI 1.23, 1.35 and 1.22, 95% CI 1.06, 1.40, respectively), while antidepressant‐only exposure was not (1.04, 95% CI 0.96, 1.12). No additive interaction was identified for combined exposure.

**Conclusions:**

Exposure to prescription opioids during pregnancy, but not antidepressants, was associated with increased relative odds of sPTB. Co‐exposure to opioids and antidepressants did not elevate the odds of sPTB above what we observed for opioid‐only exposure.

**Synopsis**

**Study question.**

This observational study aimed to investigate the association between prenatal exposure to prescription opioids, antidepressants, or their combination and spontaneous preterm birth.

**What is already known?**

The use of antidepressants and analgesic opioids medications during pregnancy has been associated with preterm birth in previous studies. Given the high prevalence of mental health disorders co‐occurring with acute pain conditions during pregnancy, antidepressants and opioids are widely prescribed during this period. However, few studies have investigated the combined exposure to both types of medication or their association with *spontaneous* preterm birth in the absence of opioid use disorder.

**What does this study add?**

Using a validated algorithm to define spontaneous preterm birth and extensive covariate adjustment, our findings suggest exposure to prescription opioids during pregnancy is a potential risk factor for spontaneous preterm birth; co‐exposure to antidepressants did not augment this risk.

## 1 BACKGROUND

Preterm delivery is a global health problem and one of the leading causes of morbidity and mortality in young children. Furthermore, the long‐term health deficits associated with preterm birth make it a leading cause of disability‐adjusted life years for children.^1^ In 2021, the US preterm birth rate was 8.8% for singleton births, with profound racial and ethnic disparities.^2^

Approximately 70% of preterm births are spontaneous,^3^ including spontaneous unexplained preterm labour with intact membranes or idiopathic preterm premature rupture of membranes (PPROM).^4^ The causes of spontaneous preterm births are multifactorial and, in most cases, cannot be clearly established.^5^ Intrauterine infection and inflammation are thought to be primary pathological factors in spontaneous preterm birth,^6^ with other obstetrical disorders also potentially implicated.^7^ Further elucidating modifiable exposures that contribute to the risk of spontaneous preterm birth is of great interest.

Medications are frequently prescribed during pregnancy despite uncertain implications for preterm delivery or other health outcomes due to a paucity of drug safety data in pregnant populations. Depression and pain conditions are two common and often co‐occurring conditions during pregnancy that are commonly treated with medications.^8^, ^9^, ^10^ Previous studies in Medicaid populations have reported that approximately 13% of pregnant patients took antidepressants^11^ and 21.6% took opioids.^12^ Established recommendations to avoid nonsteroidal anti‐inflammatory drugs for treating pain during pregnancy likely contribute to higher opioid use in this population. Preterm birth has been associated with use of antidepressants,^13^, ^14^, ^15^, ^16^, ^17^ opioids prescribed for opioid use disorder (OUD)^18^, ^19^, ^20^, ^21^ and analgesic opioids^22^, ^23^, ^24^, ^25^, ^26^ during pregnancy. However, few studies have examined concurrent exposure to these medications during pregnancy,^27^, ^28^, ^29^, ^30^ or investigated their relationship with spontaneous preterm birth specifically, distinct from indicated preterm birth. Given the prevalence of depression (11%)^31^ and acute pain (68%–72%)^32^, ^33^ in the pregnant population and potential for combined antidepressant and opioid use during pregnancy, we sought to expand the existing evidence by assessing whether prenatal exposure to antidepressants, analgesic opioids or their combination is associated with the risk of spontaneous preterm birth.

## 2 METHODS

### 2.1 Data sources

We conducted a nested case–control study in a retrospective cohort of pregnancies enrolled in the Tennessee Medicaid (TennCare) programme from 2007 to 2019. TennCare administrative records include data on Medicaid enrollment, demographics, pharmacy dispensing records and healthcare encounters and diagnoses. These administrative files and pharmacy data were linked to birth certificates and data from the Tennessee Hospital Discharge Database System, a state‐wide registry of hospital‐based encounters. Pharmacy data include prescription medication fill information, including dose and days' supply. This resource has been widely used to perform observational studies on medication safety among pregnant women enrolled in TennCare.^34^, ^35^, ^36^, ^37^

### 2.2 Study cohort and nested case–control design

**FIGURE 1:**
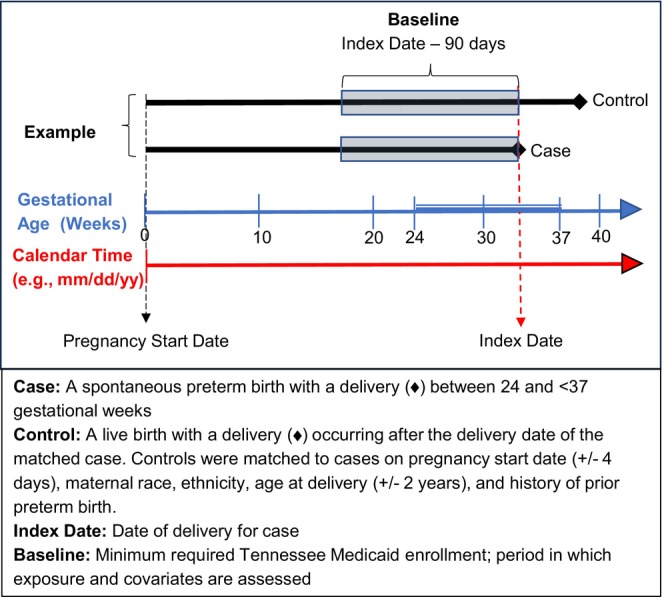
Study design.

We defined a retrospective cohort of patients enrolled in TennCare with live birth singleton deliveries between ≥24 and ≤42 weeks of gestational age, whose deliveries occurred in a Tennessee hospital between January 2007 and December 2019 and could be linked to a birth certificate. Eligible women were 15–44 years old at delivery; patients missing data on matching variables were excluded (<0.3% were missing race or ethnicity). Within this cohort, we defined spontaneous preterm birth cases as live birth deliveries that occurred ≥24 and <37 completed gestational weeks that met the criteria for spontaneous delivery based on the Klebanoff algorithm, a validated and reliable birth certificate‐based algorithm (*κ* = 0.68 compared to medical record abstraction).^38^ Eligible controls were pregnant women in the retrospective cohort who had not delivered by a case's delivery date (index date). Using incidence density sampling, we matched each case to up to 10 randomly selected controls based on estimated pregnancy start date (within 4 days), previous preterm birth (yes/no), maternal age (within 2 years), race (White, Black, Asian or other) and ethnicity (Hispanic or Non‐Hispanic), as defined in the birth certificate. Estimated pregnancy start date was calculated by subtracting the obstetric estimate of gestational age from the birth certificate from the date of delivery. Cases and controls were required to be continuously enrolled in TennCare for ≥90 days prior to the index date (baseline) (Figure 1).

**FIGURE 2:**
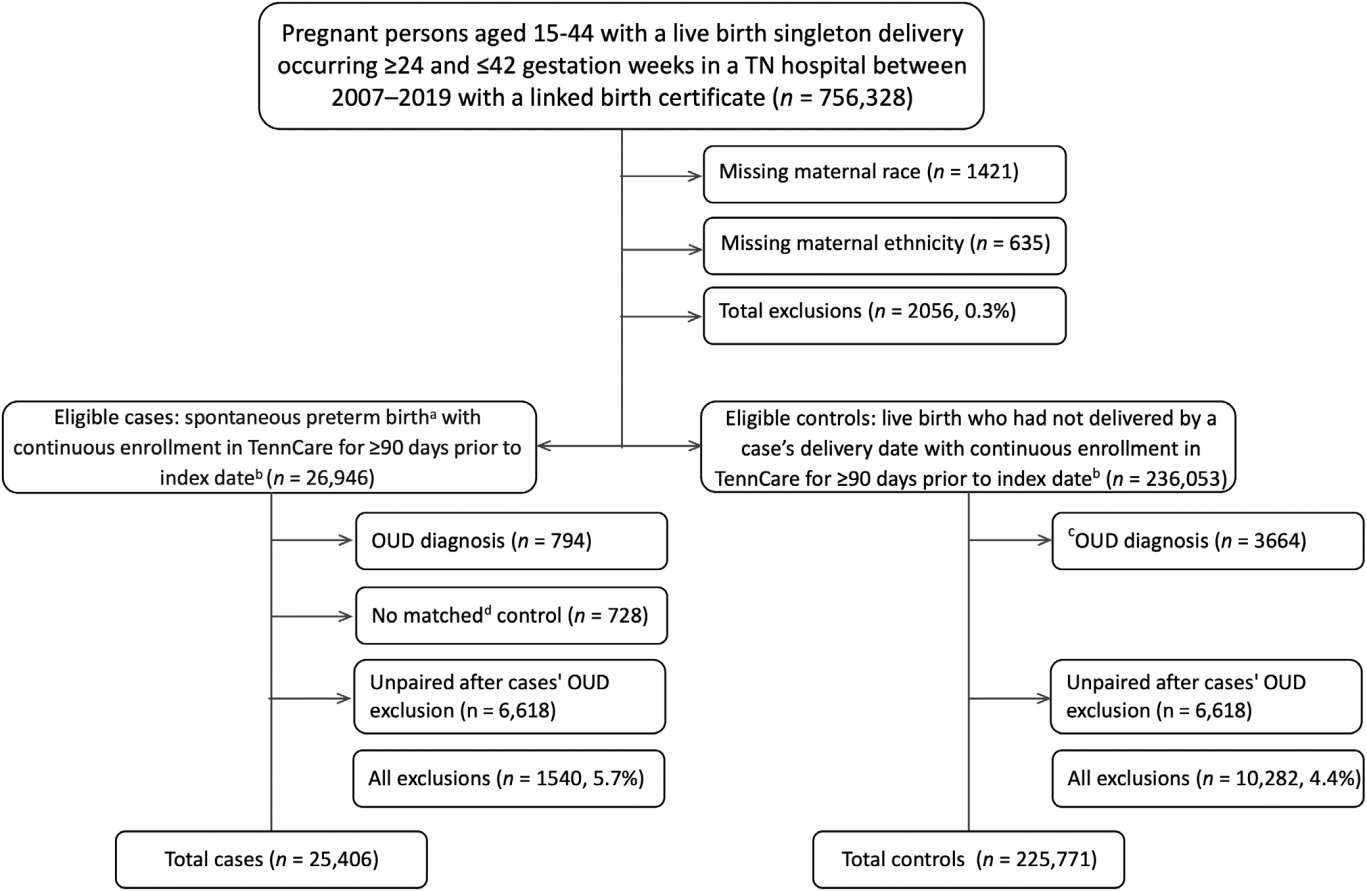
Structure of nested case–control study. OUD: Opioid use disorder.^a^ Live birth delivery that occurred 24 and <37 completed gestational weeks that met the criteria for spontaneous delivery based on a Klebanoff algorithm.^b^ Case delivery date. ^c^ identified using ICD‐9/10 codes recorded in administrative or professional claims for hospitalisation, 23‐h stay, emergency department visit or two outpatient encounters on separate dates for 90 days before delivery or women with one pharmacy dispensing in pharmacy fills in the same period of buprenorphine or buprenorphine–naloxone.^d^ Matching variables: Delivery date (within 4 days), previous preterm birth (yes/no), maternal age (within 2 years), race (White, Black, Asian or Other) and ethnicity (Hispanic or non‐Hispanic).

Because our focus was on opioids prescribed for analgesia, we excluded women with an OUD diagnosis identified during baseline using the International Classification of Diseases, Nineth (ICD‐9) and Tenth Revision (ICD‐10) codes recorded on administrative or professional claims from a hospitalisation, a 23‐h stay, emergency department visit (Table S1), two outpatient encounters on separate dates or women with ≥1 pharmacy dispensing for buprenorphine or buprenorphine–naloxone given their common use as first‐ and second‐line treatment for OUD in pregnancy.^39^, ^40^ In the event that a case was excluded on the basis of OUD diagnosis or treatment, that case's corresponding controls were also excluded (Figure 2).

### 2.3 Exposure

Exposures to prescription opioid and antidepressant medications (Table S2) were assessed using pharmacy claims. We identified the date of fill and days supplied for prescribed medications of interest during the 90‐day baseline period. We determined the number of days covered for each medication in the 60 days prior to index date (including prescriptions initiated before 60 days that extended into the 60‐day window) and defined four mutually exclusive exposure categories: (1) no days supplied for opioids or antidepressants (‘unexposed’); (2) ≥1 day covered by opioid medications with no days covered by antidepressants (‘opioid only’); (3) ≥1 day covered by antidepressant medications with no days covered by opioids (‘antidepressant only’); and (4) ≥1 of day covered by opioid and ≥1 of day covered by antidepressant medications, not necessarily overlapping (‘combined’).

In a secondary analysis, we limited antidepressant exposure to only selective serotonin reuptake inhibitors (SSRIs), given that SSRIs are the most prescribed antidepressants during pregnancy and that some reports have previously described an association with preterm birth.^16^ For this secondary analysis, other antidepressant use was reclassified into the reference group. We also performed an additional sensitivity analysis excluding other (non‐SSRI) antidepressant users from the analysis.

### 2.4 Covariates

Based on literature review^3^, ^5^ and clinical expertise, we identified and measured relevant socio‐demographic characteristics, comorbid conditions and known preterm birth risk factors considered as potential confounders between medication use during pregnancy and spontaneous preterm birth.

We defined maternal demographic characteristics using birth certificate data, including marital status, educational level, pre‐pregnancy body mass index (BMI), year of delivery and parity. Since all persons in the cohort had live birth deliveries, parity was defined as 1 for first births and >1 for those with evidence of prior live birth. Maternal comorbidities (chronic hypertension, diabetes mellitus type 2 and tobacco use) during the first and second trimesters were defined using both birth certificate data and ICD‐9/10 diagnosis/procedure codes. We defined indications for antidepressant use (depression and anxiety) and indications for opioid use (pain conditions) using ICD‐9/10 diagnosis codes. Pain conditions included abdominal and pelvic pain, urinary pain, musculoskeletal pain, myalgia, fibromyalgia, cramping, dental pain, trauma, acute pain, chronic pain, malignancy, sickle cell disease, systemic connective tissue disorders, rheumatoid arthritis, Crohn's disease and ulcerative colitis. Other maternal comorbid conditions, such as asthma and Chronic Obstructive Pulmonary Disease (COPD), as well as cerclage in the current pregnancy, were measured exclusively using ICD‐9/10 diagnosis and procedure codes. Study covariates not included in birth certificates were measured during the baseline period (Table S3).

### 2.5 Statistical analysis

We estimated odds ratios (OR and adjusted OR [aOR]) and 95% confidence intervals (CI) comparing each exposure group (opioid only, antidepressant only or combined) to the unexposed referent (no exposure to either medication) using conditional logistic regression. We examined models in three stages: (1) unadjusted, accounting for matching design variables only; (2) partially adjusted models including maternal demographics and preterm birth risk factors (maternal education level, maternal marital status, parity, cerclage in the current pregnancy and maternal comorbidities (chronic hypertension, diabetes mellitus type 2, asthma and COPD)); and (3) fully adjusted models including demographics, preterm birth risk factors and indications for medication use (diagnoses of depression, anxiety and pain conditions).

To assess joint exposure to opioids and antidepressants, we estimated additive biological interaction in the fully adjusted model. The relative excess risk due to interaction (RERI_OR_) evaluates whether the combined impact of taking opioids and antidepressants together exceeds the sum of their individual effects when measured on the OR scale.^41^ We estimated 95% CIs for each additive interaction estimate using methods described by Andersson et al.^42^ and the corresponding Excel program (Material S1). We repeated these methods for secondary analyses limited to SSRIs. We calculated E‐values^43^ using results from the conditional logistic regression in order to estimate the degree of unmeasured confounding needed to explain away our findings. We also estimated the potential influence of exposure misclassification under two hypothetical scenarios: (1) assuming characterisation of opioid use was observed with 80% sensitivity and 99% specificity, and (2) assuming characterisation of antidepressant use was observed with 95% sensitivity and 98% specificity. We performed all analyses in Stata/IC, version 17.0 (StataCorp) and Microsoft Excel.

### 2.6 Missing data

Covariate missingness ranged from 0% to 2.3% (Table 1). For primary analyses, we imputed missing values using multiple imputation by chained equations with 25 iterations. In sensitivity analyses, we generated results using a complete case analysis.

**TABLE 1:** Baseline characteristics of spontaneous preterm birth cases and matched controls from TennCare live births, 2007–2019.

	Controls	Cases
Maternal characteristics	(*n* = 225,771)	(*n* = 25,406)
Age, years^a^, mean (SD)	24.0 (5.0)	24.4 (5.4)
Ethnicity^a^, *n* (%)		
Non‐Hispanic	222,158 (98.4)	24,737 (97.4)
Hispanic	3613 (1.6)	669 (2.6)
Race^a^, *n* (%)		
White	135,650 (60.1)	15,417 (60.7)
Black or African American	89,836 (39.8)	9830 (38.7)
Asian	208 (0.1)	112 (0.4)
Other	77 (0.0)	47 (0.2)
Previous preterm birth^a^, *n* (%)	9179 (4.1)	2691 (10.6)
Year of delivery, *n* (%)		
2007	19,443 (8.6)	2348 (9.2)
2008	21,389 (9.5)	2327 (9.2)
2009	18,875 (8.4)	2091 (8.2)
2010	20,020 (8.9)	2184 (8.6)
2011	18,457 (8.2)	2056 (8.1)
2012	18,460 (8.2)	2,093 (8.2)
2013	17,406 (7.7)	1917 (7.5)
2014	15,645 (6.9)	1756 (6.9)
2015	14,600 (6.5)	1659 (6.5)
2016	14,665 (6.5)	1699 (6.7)
2017	15,429 (6.8)	1776 (7.0)
2018	14,896 (6.6)	1702 (6.7)
2019	16,486 (7.3)	1798 (7.1)
Parity, *n* (%)		
1	87,399 (39.0)	8887 (35.4)
>1	136,682 (61.0)	16,250 (64.6)
Missing	1690 (0.75)	269 (1.06)
Marital Status, *n* (%)		
Single	160,551 (71.1)	18,329 (72.2)
Married	65,104 (28.8)	7069 (27.8)
Missing	116 (0.05)	8 (0.03)
Education level, *n* (%)		
<12	49,213 (21.9)	6649 (26.3)
12	96,408 (42.8)	10,959 (43.3)
>12	79,584 (35.3)	7708 (30.4)
Missing	566 (0.25)	90 (0.35)
BMI, mean (SD)	27.2 (7.4)	25.9 (7.1)
Missing	4657 (2.06)	583 (2.3)
Tobacco use^b^, *n* (%)	56,324 (24.9)	8000 (31.5)
Depression, *n* (%)	1555 (0.7)	664 (2.6)
Anxiety, *n* (%)	1940 (0.9)	648 (2.6)
Pain conditions^c^, *n* (%)	36,796 (16.3)	5662 (22.3)
Cerclage^d^, *n* (%)	767 (0.3%)	616 (2.4%)
Chronic hypertension, n (%)	7781 (3.4%)	1598 (6.3%)
Type 2 Diabetes, *n* (%)	3054 (1.4)	782 (3.1)
COPD, *n* (%)	37 (0.0)	12 (0.0)
Asthma, *n* (%)	4712 (2.1)	1282 (5.0)

Abbreviations: BMI, body mass index; COPD, chronic obstructive pulmonary disease.

^a^ Controls were matched to cases by delivery date (within 4 days), previous preterm birth (yes/no), maternal age (within 2 years), race (White, Black, Asian or Other) and ethnicity (Hispanic or Non‐Hispanic).

^b^ Tobacco use during the first and second trimesters.

^c^ Includes abdominal and pelvic pain, urinary pain, musculoskeletal pain, myalgia, fibromyalgia, cramping, dental pain, trauma, acute pain, chronic pain, malignancy, sickle cell disease, systemic connective tissue disorders, rheumatoid arthritis, Crohn's disease and ulcerative colitis.

^d^ Cervical cerclage in the current pregnancy.

### 2.7 Ethics approval

All analytical data were anonymised, and ethical approval was granted by the Institutional Review Boards of Vanderbilt University Medical Center (190068) on 18 January 2019 and the Tennessee Department of Health (2019–0199) on 24 April 2019, who waived patient consent.

## 3 RESULTS

### 3.1 Study population

We identified 25,406 cases from a retrospective cohort of TennCare enrolees who met the initial inclusion criteria and matched these cases with 225,771 controls. Most cases (*n* = 18,769, 74%) matched to 10 controls, with the remainder matching between 1 (*n* = 741, 3%) and 9 (*n* = 2329, 9%) controls. Table 1 displays the baseline characteristics of both cases and controls in aggregate, without considering the variable ratio of cases to matched controls. In Table 1, the distribution of the matched variables was similar between the cases and controls, including mean maternal age (24.4 vs. 24 years), ethnicity (97.4% vs. 98.4% non‐Hispanic) and race (60.7% vs. 60.1% White). Cases and controls were also matched on history of preterm birth; in general, cases with prior preterm birth tended to match fewer controls (<10) than cases without prior preterm birth, so the overall frequency of prior preterm birth in controls (4.1%) appears lower than the overall frequency of prior preterm birth in cases (10.6%). Compared to controls, cases were more likely to be single and have lower levels of education. Cases also experienced higher representation of preterm birth risk factors, including a lower BMI, a higher prevalence of tobacco use during the first and second trimesters, a current cerclage, infection during pregnancy, chronic diseases and mental health conditions. Furthermore, the prevalence of depression, anxiety and pain conditions was higher in cases compared to the controls: 2.6% versus 0.7%, 2.6% versus 0.9% and 22.3% versus 16.3% respectively.

### 3.2 Antidepressant and opioid use and risk of preterm birth

Among those with opioid‐only or combined exposure in the 60 days prior to the index date, most patients filled prescriptions for hydrocodone (42% and 45%, respectively) followed by codeine (21% and 13%, respectively). The median number of days covered by opioids was 4 days (IQR: 2–8) and 6 days (IQR: 2–20) for opioid‐only or combined exposures respectively. Among pregnant patients with antidepressant‐only or combined exposure in the 60 days prior to the index date, most filled prescriptions for SSRIs (79% and 84%, respectively). The median number of days covered by an antidepressant fill was 31 days (IQR 22–52) for antidepressant‐only exposures and 30 days (IQR 22–50) for combined exposure. Opioid and antidepressant exposure was consistently higher in the case group than in the controls (Table 2). Co‐exposure to both opioids and antidepressants was not common overall but was observed more frequently in cases (1.1%) than controls (0.6%). Among those with exposure to both opioids and antidepressants, 43% experienced 5 or more days of overlapping opioid and antidepressant fills, 32% experienced 1–4 days of overlapping prescriptions and 25% had no days of overlap. The medication exposure distribution looked similar when antidepressant use was limited to SSRIs only (Table S4), as most antidepressant use in the study sample corresponded to SSRIs (79% of all antidepressant prescriptions in the controls and 78% in the cases).

**TABLE 2:** Adjusted^a^ odds ratios for spontaneous preterm birth by exposure to medication (opioids/antidepressants from Tennessee Medicaid live births, 2007–2019).

			Odds ratio (95% confidence interval)		
	Controls, *n* (%) *n* = 225,771	Cases, *n* (%) *n* = 25,406	Model 1	Model 2	Model 3
Unexposed (reference)	199,894 (88.5)	21,277 (83.7)	1.00 (Reference)	1.00 (Reference)	1.00 (Reference)
Opioid only	17,952 (8.0)	2938 (11.6)	1.51 (1.45, 1.58)	1.37 (1.31, 1.43)	1.28 (1.23, 1.34)
Antidepressant only	6464 (2.9)	899 (3.5)	1.25 (1.16, 1.34)	1.18 (1.09, 1.27)	1.05 (0.97, 1.13)
Combined	1461 (0.6)	292 (1.1)	1.81 (1.59, 2.06)	1.53 (1.34, 1.75)	1.23 (1.08, 1.41)

*Note*: Unexposed: no days supplied for opioids or antidepressants; opioid only: ≥1 day covered by opioid medications with no days covered by antidepressants; antidepressant only: ≥1 day covered by antidepressant medications with no days covered by opioids; and combined: ≥1 day covered by opioid and ≥1 day covered by antidepressant medications, not necessarily overlapping.

^a^ We adjusted by three staged models: Model 1 accounts for matching design variables; Model 2 is a partially adjusted model including maternal demographics and preterm birth risk factors (maternal education level, maternal marital status, parity, cerclage in the current pregnancy and maternal comorbidities [i.e. chronic hypertension, diabetes mellitus type 2, asthma and COPD]); and Model 3 is a fully adjusted model including demographics, preterm birth risk factors and indications for medication use (i.e. diagnoses of depression, anxiety and pain conditions). Multiple imputation (*n* = 25 iterations) was used to account for missing data.

In the unadjusted model, prenatal exposure to opioids, antidepressants or both was associated with higher odds of spontaneous preterm birth. This association attenuated slightly in the partially adjusted model and further attenuated in the fully adjusted model. In the fully adjusted model, exposure to opioids only and the combination of opioids and antidepressants were associated with the risk of spontaneous preterm birth (OR 1.28, 95% CI 1.23, 1.34; and OR 1.23, 95% CI 1.08, 1.41), respectively (*E*‐values (lower limit) = 1.88 (1.76) and 1.76 (1.37), respectively), whereas antidepressant‐only use was not associated (OR 1.05, 95% CI 0.97, 1.13) (Table 2). In the secondary analysis restricted to only SSRI antidepressants, the associations between medication exposure and spontaneous preterm birth were similar to those from the main analysis (Table S4). Likewise, results from the primary analysis did not change when we examined associations using complete case analysis (Tables S5 and S6) or when non‐SSRI antidepressant users were excluded (Table S7). Unadjusted ORs (Model 1) were similar to or lower than those generated in the exposure misclassification sensitivity analyses (Table S8). There was no evidence of additive interaction with combined opioid and antidepressant exposures overall (RERI –0.11, 95% CI –0.30, 0.08) or for opioids and SSRIs (RERI 0.00, 95% CI –0.23, 0.22).

## 4 COMMENT

### 4.1 Principal findings

In this large, nested case–control study from a Medicaid population, we observed that prescription opioid exposure alone or in combination with antidepressants was associated with increased odds of spontaneous preterm birth after adjusting for confounders, including indications for medication use. However, antidepressant (including SSRI) exposure alone was not associated with spontaneous preterm birth.

### 4.2 Strengths of the study

Primary strengths of our study include the novel study design and robust covariate adjustment. Distinct from prior studies of medication use and preterm birth, we applied a validated algorithm^38^ to identify cases of spontaneous preterm birth. Because most preterm deliveries are spontaneous with unknown aetiology, our focus on spontaneous deliveries is a unique and highly relevant contribution that was not addressed in previous studies. The nested case–control study allows for the sampling of cases and controls from the same population, matching them on pregnancy start date ensures comparable gestational age of exposure, effectively mitigating temporal and biological confounding. Additional strengths include the use of birth certificate information to supplement other sources of data, including detailed characterisation of pregnancy and delivery events. Furthermore, using linked administrative and pharmacy files and data from the Tennessee Hospital Discharge Database System and vital records allows a complete assessment of exposure and covariate variables.

### 4.3 Limitations of the data

Our study also has some limitations. First, our findings could have been influenced by exposure misclassification. While pharmacy records are a widely used, reliable source of medication data,^44^, ^45^ including during pregnancy,^46^, ^47^ we acknowledge that measurement of prescription fills is distinct from measurement of actual medication use. Furthermore, we were unable to assess the use of nonprescription medications (e.g. illicit opioids) or medications accessed outside of Medicaid (e.g. cash payments). It is unlikely that misclassification would differ by case status (nondifferential), yet due to the polytomous nature of our exposure definition, it is not possible to intuitively predict the direction of bias.^48^ However, when we examined two plausible scenarios of misclassification, our results were conservative in comparison.

Second, although we controlled for potential confounders including common indications for use of prescription opioids and antidepressants, we could not measure the severity of clinical conditions which may introduce residual confounding. Other sources of residual confounding may include unmeasured sociodemographic factors, behaviours (e.g., alcohol and diet) and use of other medications such as benzodiazepines that may have been used outside of the common conditions we accounted for in our analysis. An unmeasured confounder associated with opioid use and spontaneous preterm birth at a magnitude of 1.37‐ to 1.76‐fold could explain away the lower limits of our OR confidence intervals for primary opioid and joint exposure findings.

We further note modest heterogeneity in our definition of combined exposure, such that it includes some patients who were exposed to medications on the same day as well as some patients who were exposed to medications on different days in the same 60‐day window. Given the extensive half‐life of certain antidepressants,^49^, ^50^ this design feature does not detract from our overall findings. However, future studies in this area may consider how the window of overlapping medication use may influence findings. Finally, our study focused on a Medicaid population within Tennessee, and although the programme provides healthcare coverage to about 50% of births in the state and similar proportions in other states,^51^ our findings may not directly apply to pregnant individuals in other geographic locations or those with private insurance.

### 4.4 Interpretation

Contemporary literature has extensively explored the independent associations between opioid and antidepressant use and preterm birth. However, prior studies of antidepressant use^13^, ^14^, ^15^, ^16^ typically do not distinguish spontaneous preterm birth from indicated preterm birth, as we did in this analysis. Furthermore, these studies have implemented varying approaches to control for the presence or severity of depression, which our study suggests is an important confounder. Opioids have been associated with preterm birth in prior studies, including a large Swedish retrospective cohort study.^52^ However, these results attenuated when alternative study designs such as unexposed sibling comparison were implemented, and authors concluded that findings were likely driven by unmeasured confounding related to factors such as pain indications or pain severity. Consistent with our finding, Esposito et al.^24^ found an increased risk of preterm birth among those who were dispensed ≥2 opioids at any time in pregnancy after adjusting for maternal demographics, comorbidities and medications (including antidepressants) in a nationwide Medicaid sample.

We identified two prior studies that assessed joint exposure to antidepressants and opioids during pregnancy and preterm birth. Kallen et al.^28^ reported an increased risk of preterm birth with SSRI‐only dispensing (aOR 1.38, 95% CI 1.19, 1.61). Risk markedly increased with combined dispensing for SSRI and opioids (aOR 2.44, 95% CI 1.89, 3.52), however, the independent use of opioids was not assessed. In a large Swedish population cohort, researchers found a higher risk of preterm birth among pregnant women prescribed opioids (RR 1.27, 95% CI 1.22, 1.33), SSRIs (RR 1.34, 95% CI 1.27, 1.42) and both in combination (RR 1.70, 95% CI 1.47, 1.96) compared to unexposed women.^30^ This study's findings differed from ours in that they observed an increased risk for preterm birth with SSRIs alone (not distinguishing spontaneous from indicated), but consistent with our findings, the study observed no evidence of additive interaction due to joint exposure to SSRIs and opioids (RERI 0.09, 95% CI –0.17, 0.34).

The association between both opioids and antidepressants and preterm birth is biologically plausible. Antidepressants and opioids can cross the placenta and alter the placental barrier.^24^, ^53^, ^54^, ^55^, ^56^ Opioids, in particular, may also contribute to spontaneous preterm birth via pathways involving maternal infection. During pregnancy, studies have shown an affectation in placental permeability with the use of methadone, which could impair placental barrier function and allow pathogens to cross.^57^ Those observations are further supported by studies that have found a higher risk of infection in opioid‐prescribed patients.^58^, ^59^, ^60^

## 5 CONCLUSIONS

Spontaneous preterm birth is a common adverse obstetric outcome and one of the leading causes of infant mortality and morbidity. Our study found an association between opioid exposure and spontaneous preterm birth, and that antidepressants (primarily SSRIs) were not associated with spontaneous preterm birth. We did not find evidence that co‐exposure to antidepressants and opioids increases the risk of spontaneous preterm birth above and beyond the increased risk observed from opioid exposure alone. Clinicians should consider these findings relative to the risk of untreated maternal mood disorders and pain when deciding whether to prescribe opioids and antidepressants during pregnancy.

## AUTHOR CONTRIBUTIONS

M.A.A., C.G.G. and S.S.O. contributed to conception of the research question and study design. M.C.P.‐A. performed the analysis, with support from M.A.A. All authors contributed to the interpretation of results. The manuscript was drafted by M.C.P.‐A. and critically revised by all authors. All authors approved this version of the manuscript to be published and agree to be accountable for all aspects of the work (M.C.P.‐A., S.S.O., O.B., A.W., A.P., A.A.L., A.J.S., C.G.G. and M.A.A.).

## FUNDING INFORMATION

This study was supported by the National Institutes of Health (NICHD; R21HD104983). A.D.W. was supported by K01DA051683 from the National Institute on Drug Abuse. S.S.O. was supported by K23DA047476 from the National Institute on Drug Abuse. A.A.L. was supported by K01DA050740 from the National Institute on Drug Abuse. The funders of the study had no role in study design, collection, analysis or interpretation of data, writing of the report or decision to submit the paper for publication.

## CONFLICT OF INTEREST STATEMENT

C.G.G. has received consultant fees from Merck and received research support from Syneos Health, NIH, CDC, FDA and AHRQ. All other authors declare no conflicts of interest.

## Supporting information


Data S1.

## Data Availability

The data that support the findings of this study are protected under a data use agreement and cannot be shared by investigators.
