# Supplementary material for: Opioid analgesic and antidepressant use during pregnancy and the risk of spontaneous preterm birth: A nested case–control study
Source: Paediatr Perinat Epidemiol. Author manuscript; Available in PMC 2025 Feb 1. (PMC11781513; doi:10.1111/ppe.13142)
Supplement: Supplementary Material [file NIHMS2045266-supplement-Supplementary_Material.docx]

**Supplemental Material**

Opioid Analgesic and Antidepressant Use During Pregnancy and the Risk of Spontaneous Preterm Birth: A Nested Case-Control Study

Padilla-Azain et al.

**eMaterial 1.** Excel program for calculation of relative excess risk of interaction

<https://www.biostatistik.se/epinetcalculation.xls>

from Andersson T, Alfredsson L, Källberg H, Zdravkovic S, Ahlbom A. Calculating measures of biological interaction. Eur J Epidemiol. 2005;20(7):575-9. doi: 10.1007/s10654-005-7835-x. PMID: 16119429.

**eTable 1.** ICD-9/10 codes used to define OUD on administrative or professional claims.

| Diagnosis | ICD- 9 codes | ICD-10 codes |
| --- | --- | --- |
|  | 304.00, 304.01, 304.02,304.03, 304.70, 304.71,304.72,304.73, 305.5, 305.50, 305.51, 305.52, 305.53, 965.0, 965.00, 965.01, 965.02, 965.09, 970.1, E850.0, E850.1, E850.2, E935.0, E935.1 | F11.1, F11.10, F11.12, F11.120, F11.121, F11.122, F11.129, F11.14, F11.15, F11.150, F11.151, F11.159, F11.18, F11.181, F11.182, F11.188, F11.19, F11.2, F11.20, F11.21, F11.220, F11.221, F11.22, F11.222, F11.229, F11.23,F11.24, F11.25, F11.250, F11.251, F11.259, F11.28, F11.281, F11.282, F11.288, F11.29, F11.90, F11.920, F11.921, F11.922, F11.929, F11.93, F11.94, F11.950, F11.951, F11.959, F11.981, F11.982, F11.988, F11.99, T40.0X1A, T40.0X2A, T40.0X3A , T40.0X4A, T40.1X1A, T40.1X2A, T40.1X3A, T40.1X4A, T40.2X1A, T40.2X2A, T40.2X4A, T40.3X1A, T40.3X2A, T40.3X4A, T40.3X5A, T40.4X1A, T40.4X2A , T40.4X3A, T40.4X4A, T40.411A, T40.412A, T40.413A, T40.414A, T40.415A , T40.421A, T40.422A, T40.423A , T40.424A, T40.425A, T40.491A, T40.492A , T40.493A, T40.494A, T40.495A, T40.601A, T40.602A, T40.603A, T40.604A, T40.691A, T40.692A, T40.693A, T40.694A, T40.0X1D, T40.0X1S, T40.0X4D, T40.0X4S, T40.1X1D, T40.1X1S, T40.1X4D, T40.1X4S, T40.2X1D, T40.2X1S, T40.2X4D, T40.2X4S, T40.3X1D, T40.3X1S, T40.3X4D, T40.3X4S |
|  |  |  |
|  |  |  |
| OUD |  |  |
|  |  |  |

ICD9/10: International Classification of Diseases, Nineth (ICD-9) and Tenth Revision (ICD-10). OUD: Opioid Use Disorder

**eTable 2.** Antidepressants and analgesics opioids included as exposure variables.

| Group Name | | Medication |
| --- | --- | --- |
| Opioids | Agonist Opioids | Propoxyphene and Propoxyphene, Codeine, Meperidine HCL, Morphine Sulfate, Dihydrocodeine Combinations, Hydrocodone Combinations, Hydromorphone, Oxycodone Combinations, Oxymorphone Hydrochloride, Levorphanol, Fentanyl, Tramadol Hydrochloride, Meperidine-Promethazine, Tapentadol, Meperidine HCL, Morphine-Naltrexone |
|  | Mixed agonist-antagonist opioids | Pentazocine Hydrochloride, Butorphanol Tartrate, Nalbuphine Hydrochloride, Pentazocine-Naloxone |
|  | Opiates and barbiturates | Codeine-Butalbital |
|  | Narcotic-NSAID combinations | Hydrocodone-Ibuprofen |
| Antidepressants | Miscellaneous Antidepressant | Trazodone hydrochloride, Bupropion hydrochloride, Nefazodone, Mirtazapine, Vortioxetine, Esketamine, Solriamfetol HCL |
|  | Tricyclics | Amitriptyline hydrochloride, Desipramine, Doxepin hydrochloride, Imipramine, Nortriptyline hydrochloride, Protriptyline hydrochloride, Trimipramine maleate, Amoxapine, Maprotiline hydrochloride, Clomipramine hydrochloride |
|  | SSRI | Fluoxetine hydrochloride, Sertraline hydrochloride, Paroxetine hydrochloride, Fluvoxamine, Citalopram, Escitalopram, Vilazodone hydrochloride |
|  | SNRI | Atomoxetine, Milnacipran, Levomilnacipran |
|  | SSNRI | Venlafaxine, Duloxetine HCL, Desvenlafaxine succinate |

HCL = hydrochloride. NSAID: Nonsteroidal Anti-inflammatory Drug. SSRI: Selective Serotonin Reuptake Inhibitor. SNRI: Serotonin - Norepinephrine Reuptake Inhibitors. SSNRI: Selective Serotonin – Norepinephrine Reuptake Inhibitor

**eTable 3.** ICD-9/10 and CPT/HCPCS codes used to define covariates on administrative or professional claims.

| Covariates | Diagnosis | ICD 9 - codes | ICD - 10 codes | CPT/HCPCS |
| --- | --- | --- | --- | --- |
| Cerclage^a^ | Cervical cerclage | 654.5, 67.51 | O34.3 | 59320 |
| Tobacco use^b^ | Tobacco | 649.0, 305.1 | 649.0, 305.1 |  |
|  | Chronic hypertension | 642.0, 642.1, 642.2, 642.7, 401, 403, 404, 405 | O10, O11, I10, I12, I13, I15, I16 |  |
| Maternal comorbidities | Type 2 diabetes | 250 | O24.1, E11 |  |
|  | Asthma | 493 | O99.5, J45 |  |
|  | COPD | 491.2 | J44 |  |
|  | Depression | 311 | F32 |  |
| Maternal mental health disorders | Anxiety | 300 | F4.1 |  |
|  | Abdominal and pelvic pain | 789.0, 789.3, 789.4, 789.6, 789.7 | R10 |  |
|  | Urinary pain | 788.0, 788.1, 788.2 | N20, N21, N22, N23, R30, R39.89 |  |
|  | Musculoskeletal pain | 724, 719.4 | M54, M25.5 |  |
|  | Myalgia, fibromyalgia, cramping | 729.1, 729.82 | M79.1, M79.7, R25.2 |  |
|  | Dental pain | 525.8 | K09.9 |  |
|  | Trauma | 338.1 | G89.111 |  |
| Pain conditions | Acute pain | 338.19 | G89.1 |  |
|  | Chronic pain | 338.0, 338.2, 338.4 | G89.0, G89.2, G89.4 |  |
|  | Malignancy | 338.3 | G89.3 |  |
|  | Sickle cell disease | 282.41, 282.42, 282.6 | D57.0, D57.1, D57.2, D57.4, D57.8 |  |
|  | Systemic connective tissue disorders | 710 | M30, M31, M32, M33, M35, M36 |  |
|  | Rheumatoid arthritis | 714 | M06 |  |
|  | Colitis (Crohn’s disease, ulcerative colitis) | 555, 556 | K50, K51 |  |

ICD9/10: International Classification of Diseases, Nineth (ICD-9) and Tenth Revision (ICD-10). CPT/HCPCS: Current Procedural Terminology/Healthcare Common Procedure Coding System. COPD: Chronic Obstructive Pulmonary Disease.

^a^ Cervical cerclage in the current pregnancy.

^b^ Tobacco use during the first and second trimesters.

**eTable 4.** Adjusted^a^ odds ratios for spontaneous preterm birth by exposure to medication (opioids / SSRIs) from Tennessee Medicaid live births, 2007-2019..

|  |  |  |  | OR (95% CI) |  |
| --- | --- | --- | --- | --- | --- |
|  | Controls  *n* (%)  n = 225,771 | Cases  *n* (%)  n = 25,406 | Model 1 | Model 2 | Model 3 |
| Reference | 201,211 (89.1) | 21,472 (84.5) | 1.00 (Reference) | 1.00 (Reference) | 1.00 (Reference) |
| Opioid only | 18,325 (8.1) | 3002 (11.8) | 1.51 (1.45, 1.58) | 1.37 (1.31, 1.43) | 1.29 (1.22, 1.33) |
| SSRI only | 5,147 (2.3) | 704 (2.8) | 1.25 (1.16, 1.34) | 1.17 (1.08, 1.28) | 1.04 (0.95, 1.13) |
| Combined | 1,088 (0.5) | 228 (0.9) | 1.81 (1.59, 2.06) | 1.63 (1.40, 1.89) | 1.31 (1.12, 1.52) |

OR: Odds Ratio. 95% CI: 95% Confidence Interval.

SSRIs: Selective Serotonin Reuptake Inhibitors.

^a^ Model 2 is a partially adjusted model including maternal demographics and preterm birth risk factors (maternal education level, maternal marital status, parity , cerclage in the current pregnancy, maternal comorbidities (i.e., chronic hypertension, diabetes mellitus type 2, asthma, and COPD); Model 3 is a fully adjusted model including demographics, preterm birth risk factors and indications for medication use (i.e., diagnoses of depression, anxiety and pain conditions). Reference: no days supplied for opioids or SSRIs (this group includes those with ≥ 1 day of non-SSRI antidepressants); Opioid only: ≥1 day covered by opioid medications with no days covered by SSRIs; SSRI only: ≥1 day covered by SSRI medications with no days covered by opioids; Combined: ≥1 of day covered by opioid and ≥1 of day covered by SSRI medications, not necessarily overlapping. Multiple imputation (n = 25 iterations) was used to account for missing data.

**eTable 5.** Adjusted^a^ odds ratios (OR) for spontaneous preterm birth by exposure to medication (opioids / antidepressants) from Tennessee Medicaid live births, 2007-2019, complete case analysis

|  |  | OR (95% CI) |  |
| --- | --- | --- | --- |
|  | Model 1 | Model 2 | Model 3 |
| Unexposed (Reference) | 1.00 (Reference) | 1.00 (Reference) | 1.00 (Reference) |
| Opioid only | 1.51 (1.45, 1.58) | 1.38 (1.32, 1.44) | 1.29 (1.23, 1.35) |
| Antidepressant only | 1.25 (1.16, 1.34) | 1.18 (1.09, 1.27) | 1.04 (0.96, 1.12) |
| Combined | 1.81 (1.59, 2.06) | 1.51 (1.32, 1.73) | 1.22 (1.06, 1.40) |

OR: Odds Ratio. CI: Confidence Interval.

Unexposed: no days supplied for opioids or antidepressants; Opioid only: ≥1 day covered by opioid medications with no days covered by antidepressants; Antidepressant only: ≥1 day covered by antidepressant medications with no days covered by opioids; Combined: ≥1 of day covered by opioid and ≥1 of day covered by antidepressant medications, not necessarily overlapping. ^a^ We adjusted by three staged models: Model 1 accounts for matching design variables; Model 2 is a partially adjusted model including maternal demographics and preterm birth risk factors (maternal education level, maternal marital status, parity , cerclage in the current pregnancy, maternal comorbidities (i.e., chronic hypertension, diabetes mellitus type 2, asthma, and COPD); Model 3 is a fully adjusted model including demographics, preterm birth risk factors and indications for medication use (i.e., diagnoses of depression, anxiety and pain conditions).

**eTable 6.** Adjusted^a^ odds ratios for spontaneous preterm birth by exposure to medication (opioids / SSRIs) in each staged model from Tennessee Medicaid live births, 2007-2019, complete case analysis.

| OR (95% CI) | | | |
| --- | --- | --- | --- |
|  | Model 1 | Model 2 | Model 3 |
| Reference | 1.00 (Reference) | 1.00 (Reference) | 1.00 (Reference) |
| Opioid only | 1.51 (1.45, 1.58) | 1.37 (1.31, 1.43) | 1.28 (1.23, 1.34) |
| SSRIs only | 1.23 (1.13, 1.33) | 1.17 (1.08, 1.28) | 1.04 (0.95, 1.13) |
| Combined | 1.91 (1.65, 2.21) | 1.64 (1.41, 1.91) | 1.31 (1.12, 1.53) |

OR: Odds Ratio. 95% CI: 95% Confidence Interval. SSRIs: Selective Serotonin Reuptake Inhibitors. Reference: no days supplied for opioids or SSRIs (this group includes those with ≥ 1 day of non-SSRI antidepressants); Opioid only: ≥1 day covered by opioid medications with no days covered by SSRIs; SSRI only: ≥1 day covered by SSRI medications with no days covered by opioids; Combined: ≥1 of day covered by opioid and ≥1 of day covered by SSRI medications, not necessarily overlapping. ^a^ We adjusted by three staged models: Model 1 accounts for matching design variables; Model 2 is a partially adjusted model including maternal demographics and preterm birth risk factors (maternal education level, maternal marital status, parity , cerclage in the current pregnancy, maternal comorbidities (i.e., chronic hypertension, diabetes mellitus type 2, asthma, and COPD); Model 3 is a fully adjusted model including demographics, preterm birth risk factors and indications for medication use (i.e., diagnoses of depression, anxiety and pain conditions).

**eTable 7.** Adjusted^a^ odds ratios for spontaneous preterm birth by exposure to medication (opioids / SSRIs) in each staged model from Tennessee Medicaid live births, 2007-2019 , excluding non-SSRI antidepressant users.

|  |  |  |  | OR (95% CI) |  |
| --- | --- | --- | --- | --- | --- |
|  | Controls  *n* (%)  *n* = 224,081 | Cases  *n* (%)  *n* = 25,147 | Model 1 | Model 2 | Model 3 |
| Reference | 199,894 (89.2) | 21,277 (84.6) | 1.00 (Reference) | 1.00 (Reference) | 1.00 (Reference) |
| Opioid only | 17,952 (8.0) | 2,938 (11.7) | 1.51 (1.45, 1.58) | 1.37 (1.31, 1.44) | 1.28 (1.23, 1.35) |
| SSRI only | 5,147 (2.3) | 704 (2.8) | 1.24 (1.14, 1.34) | 1.18 (1.08, 1.28) | 1.04 (0.95, 1.13) |
| Combined | 1,088 (0.5) | 228 (0.9) | 1.92 (1.66, 2.23) | 1.64 (1.41, 1.91) | 1.31 (1.12, 1.53) |

N = 1949 (1,690 controls and 259 cases) with non-SSRI exposure excluded. Reference: no days supplied for opioids or SSRIs; Opioid only: ≥1 day covered by opioid medications with no days covered by SSRIs; SSRI only: ≥1 day covered by SSRI medications with no days covered by opioids; Combined: ≥1 of day covered by opioid and ≥1 of day covered by SSRI medications, not necessarily overlapping. OR: Odds Ratio. 95% CI: 95% Confidence Interval. SSRIs: Selective Serotonin Reuptake Inhibitors. Reference: no days supplied for opioids or SSRIs; Opioid only: ≥1 day covered by opioid medications with no days covered by SSRIs; SSRI only: ≥1 day covered by SSRI medications with no days covered by opioids; Combined: ≥1 of day covered by opioid and ≥1 of day covered by SSRI medications, not necessarily overlapping. ^a^ We adjusted by three staged models: Model 1 accounts for matching design variables; Model 2 is a partially adjusted model including maternal demographics and preterm birth risk factors (maternal education level, maternal marital status, parity , cerclage in the current pregnancy, maternal comorbidities (i.e., chronic hypertension, diabetes mellitus type 2, asthma, and COPD); Model 3 is a fully adjusted model including demographics, preterm birth risk factors and indications for medication use (i.e., diagnoses of depression, anxiety and pain conditions).

**eTable 8.** Odds ratios for spontaneous preterm birth by exposure to medication (opioids / antidepressants) under exposure misclassification scenarios

|  | Controls  *n* (%)  n = 225,771 | Cases  *n* (%)  n = 25,406 | OR |
| --- | --- | --- | --- |
| Observed exposure distribution | | | |
| Reference | 199,894 (88.5) | 21,277 (83.7) | 1.00 (Reference)* |
| Opioid only | 17,952 (8.0) | 2,938 (11.6) | 1.51 (1.45, 1.58)* |
| Antidepressant only | 6,464 (2.9) | 899 (3.5) | 1.25 (1.16, 1.34)* |
| Combined | 1,461 (0.6) | 292 (1.1) | 1.81 (1.59, 2.06)* |
| “True” exposure distribution and OR if observed opioids were misclassified with 80% sensitivity and 99% specificity | | | |
| Reference | 206,860 (91.6) | 22,061 (86.8) | 1.0 (Ref) |
| Opioid only | 11,510 (5.0) | 2,225 (8.7) | 1.81 (1.73, 1.90) |
| Antidepressant only | 6,464 (2.8) | 899 (3.5) | 1.30 (1.21, 1.40) |
| Combined | 937 (0.4) | 221 (0.8) | 2.20 (1.90, 2.57) |
| “True” exposure distribution and OR if observed antidepressants were misclassified with 95% sensitivity and 98% specificity | | | |
| Reference | 204000 (90) | 21740 (86) | 1.0 (Ref) |
| Opioid only | 17,952 (8) | 2,938 (12) | 1.52 (1.47, 1.60) |
| Antidepressant only | 3115 (1.7) | 550 (2) | 1.66 (1.51, 1.82) |
| Combined | 704 (0.3) | 178 (0.7) | 2.37 (2.00, 2.81) |

*Corresponds to Model 1 in Table 2
